# Zero-fluoroscopy catheter ablation for arrhythmias in pregnancy: validation of a standardized approach with 5-year follow-up

**DOI:** 10.3389/fcvm.2026.1859227

**Published:** 2026-08-27

**Authors:** Sebastian Stec, Marek Bronisz, Joanna Kornaszewska, Katarzyna Styczkiewicz, Marcin Nosal, Alicja Hajduk-Hejnar, Anna Kustroń, Szymon Stec, Agata Bielecka-Dąbrowa, Marta Kornaszewska, Janusz Śledź

**Affiliations:** 1 Department of Cardiac Surgery and Transplantology, National Medical Institute of the Ministry of Interior and Administration and Institute for Cardiovascular Science, CardioNeuroLab, CardioMedicum, Kraków, Poland; 2 Pathology Department, University of Opole, Opole, Poland; 3 Private Cardiology Practice, Kleczany, Poland; 4 Department of Cardiology, Faculty of Medicine Collegium Medicum University of Rzeszów, Rzeszów, Poland; 5 Centrum Kardiologii Inwazyjnej, Elektroterapii I Angiologii w Krośnie; Intercard Sp. z o. o., Krosno, Poland; 6 Department of Anaesthesiology and Intensive Care, Subcarpathian Voivodeship Hospital, Krosno, Poland; 7 Biosphere Research and Clinical Trial, Rzeszów, Poland; 8 Elmedica, EP-Network, Skarżysko-Kamienna, Poland; 9 Department of Cardiology and Congenital Diseases of Adults, Polish Mother's Memorial Hospital Research Institute (PMMHRI), Lodz, Poland; 10 Department of Preventive Cardiology and Lipidology, Medical University of Lodz, Łódź, Poland

**Keywords:** ablation, arrhythmia, catheter, pregnancy, zero-fluoroscopy

## Abstract

**Background:**

Increasing experience with zero- (ZF) or near-zero fluoroscopy catheter ablation (CA) supports the implementation of an early fluoroless approach for recurrent, symptomatic arrhythmias during pregnancy.

**Aim:**

This study evaluated the feasibility, efficacy, and safety of CA using a standardized ZF approach during pregnancy.

**Methods:**

Data were derived from a large, prospective multicenter registry (ELEKTRO-RARE-A-CAREgistry). Between 2012 and 2019, more than 2,655 CA procedures were performed in women using an intention-to-treat ZF approach. The procedures were performed using (1) femoral access, (2) a double-catheter technique without intracardiac echocardiography, (3) an electroanatomic mapping system for mapping and navigation, and (4) conscious light sedation. A shared decision-making approach was applied, including consultation with a pregnancy heart team.

**Results:**

The study group consisted of 18 consecutive pregnant women without structural heart disease (mean age: 30.6 ± 4.7 years; range: 20–38 years). The mean gestational age at the time of CA was 20.2 ± 9.5 weeks (range: 7–37 weeks), representing <0.01% of all female patients referred for CA. During 18 index procedures, a total of 21 arrhythmogenic substrates were ablated. The major indications for CA during pregnancy were atrioventricular nodal reentrant tachycardia (*n* = 10), orthodromic atrioventricular reentrant tachycardia (*n* = 2), focal idiopathic ventricular arrhythmia (*n* = 5), atrial tachycardia (*n* = 2), and atrial fibrillation (AF; *n* = 1). In the patient with AF, general anesthesia and transesophageal echocardiography were used to guide ZF transseptal puncture and isolation of the right pulmonary veins, where focal AF triggers were identified. All the procedures were successfully completed without fluoroscopy and without serious maternal or fetal complications. The mean procedure and ablation application times were 51.5 ± 6.8 min and 394 ± 338 s, respectively. In one patient, CA of a second premature ventricular contraction focus was postponed until after delivery. After a 5-year follow-up, no adverse effects related to CA performed during pregnancy were observed in the children, and CA was effective in 80% of the women.

**Conclusions:**

The implementation of a pregnancy heart team and a standardized fluoroless protocol for CA in routine electrophysiological practice enables safe and effective ablation of maternal supraventricular tachycardia and idiopathic ventricular arrhythmias during pregnancy.

## 1 Introduction

The higher incidence of cardiac tachyarrhythmias during pregnancy is associated with hemodynamic, hormonal, autonomic, and emotional changes occurring throughout gestation. The most common is supraventricular tachycardia (SVT), presenting *de novo* or as an exacerbation of a pre-existing arrhythmia, and is reported in approximately 1%–2% of pregnancies (1–4).

Arrhythmia is one of the most common indications for non-elective cardiovascular hospitalization during pregnancy (5, 6). Although the majority of maternal arrhythmias during pregnancy are benign, some are highly symptomatic and require intensive treatment, including non-pharmacological maneuvers, antiarrhythmic drugs, or, in drug-refractory cases, catheter ablation (CA) (1–4, 7).

During episodes of tachyarrhythmia, maneuvers that increase vagal tone (e.g., the Valsalva maneuver or carotid sinus massage) or intravenous antiarrhythmic drugs (adenosine, beta-blockers) are recommended before direct external electrical cardioversion (1–3). Long-term management of pregnant women with symptomatic arrhythmias is more complex and particularly challenging during the first trimester, when discontinuation of all antiarrhythmic drugs is recommended whenever possible.

Advances in interventional cardiac electrophysiology (EP) have led to the incorporation of zero-fluoroscopy (ZF) or near-ZF approaches for CA of SVT and ventricular tachyarrhythmias (8–18, 34). The implementation of three-dimensional electroanatomical mapping systems allows real-time visualization and navigation of catheters, enabling the creation of virtual cardiac anatomy and precise localization of critical sites within the arrhythmia substrate (11, 14, 19). Advanced echocardiographic techniques, including transesophageal and intracardiac echocardiography, enable guidance of the transseptal approach and navigation of sheaths and catheters (11, 19). Consequently, fluoroless CA of cardiac arrhythmias has become a routine procedure performed by experienced operators in selected patients.

The growing experience with ZF or near-ZF CA, demonstrating high success rates and a minimal risk of periprocedural complications, supports the use of these procedures during pregnancy, when radiation exposure may pose potential risks to both the mother and the fetus (1, 12, 20–25). However, data on perinatal outcomes and results of ZF CA in pregnant patients remain limited. Therefore, the aim of this study was to evaluate the feasibility, efficacy, and safety of treating refractory arrhythmias during pregnancy after the implementation of a standardized ZF-CA approach within one of the largest ZF CA registries.

## 2 Materials and methods

The data were derived from a prospective registry (ELEKTRO-RARE-A-CAREgistry) covering all electrophysiological studies (EPS) and CA procedures performed in 10 Polish EP centers between 2010 and 2019. In 2012 and 2013, the ZF and near-ZF protocols were successfully implemented by two expert operators. From 2014 onward, the procedures were performed by three experienced EP operators employed as consultants in 10 cardiological departments who had each completed at least 100 ZF ablations as the primary operator, which was considered the learning-curve threshold. A database containing medical records from approximately 7,000 EPS and CA procedures performed between January 2012 and September 2019 was analyzed. During this period, 2,655 CA procedures were performed in women using an intention-to-treat ZF approach.

The study group consisted of 18 pregnant women scheduled for ablation during pregnancy due to drug-refractory or poorly tolerated arrhythmia, in accordance with current guidelines (4, 8–10) and following consensus of the pregnancy heart team that the arrhythmia posed a potential risk to both the mother and the fetus. The medical records of the study group were retrieved, and the following data were analyzed: age, comorbidities, indication for the ablation procedure, vascular access, ablation duration, number of radiofrequency applications, and total duration of radiofrequency energy delivery. In addition, data on gravidity, gestational age at the time of the ablation procedure, and previous history and treatment of arrhythmia were assessed. Information regarding child development was collected during long-term telephone follow-up with the mothers, with particular attention paid to developmental abnormalities reported by the parents. Perinatal outcomes were assessed using obstetric records, prenatal ultrasound examinations, and neonatal evaluation, including Apgar scores. All fetuses underwent ultrasound examination immediately before the catheter ablation procedure.

Long-term follow-up assessment was performed between 2019 and 2023 to obtain at least 1 year of observation after CA during pregnancy. Follow-up information was collected through telephone interviews, with particular attention to arrhythmia recurrence, use of antiarrhythmic drugs after CA, and child development. All patients provided written informed consent for the procedure and study protocol. The study was approved by an institutional review board.

### 2.1 Catheter ablation procedure

The fluoroscopy system was positioned away from the patient table, and laboratory staff did not wear lead protection. An EnSite NavX system (St. Jude Medical, St. Paul, MN, USA) was used for non-fluoroscopic mapping and catheter navigation during CA. All the patients remained conscious and received either very light sedation or no sedation. The procedures were performed via femoral access using a double-catheter technique without intracardiac echocardiography. A standardized two-catheter approach was employed to minimize additional venous punctures and mitigate the risk of catheter dislodgement. The operators were familiar with this approach. The presence of a right-sided accessory pathway was excluded through baseline and final decremental right ventricular pacing, right ventricular overdrive pacing during tachycardia, and mapping of the earliest retrograde atrial activation during sustained narrow QRS complex tachycardia. In one case of atrial fibrillation (AF), general anesthesia and transesophageal echocardiography were used to guide ZF transseptal puncture and right-sided pulmonary vein isolation. ICE is not reimbursed in Poland and we reported our clinical practice for physicians who do not perform CA with Intracardiac echocardiography (ICE). Thrombotic risk was minimized through the administration of a baseline prophylactic intravenous bolus of 3,000 units of unfractionated heparin to prevent catheter-associated thrombosis. An additional bolus was administered when left-sided access was required. Postprocedural care involved the administration of low-molecular-weight heparin (LMWH) based on the obstetrician's decision or if an atrial fibrillation ablation had been performed. During the patients’ hospital stay, prophylactic LMWH was administered, with an additional 75 mg of aspirin (ASA) for 4 weeks afterwards. The patients were mobilized after 8–12 h depending on vascular vein-only access (8 h) or arterial access (12 h) for a retrograde approach. Procedure time was defined as the interval from the first femoral puncture to catheter removal at the end of the ZF ablation procedure. All information regarding periprocedural parameters was prospectively collected by qualified and experienced EP nurses and/or EP fellows.

Obstetric assessment and fetal echocardiography were performed before and after the procedure (26). Perioperative complications were also evaluated. Following the ablation procedure, antiarrhythmic medications were discontinued in all patients.

### 2.2 Statistical analysis

Categorical variables are described using counts and percentages. Quantitative variables are described using means and standard deviations. All statistical analyses were performed using R 3.4 (R Foundation for Statistical Computing, Vienna, Austria).

## 3 Results

**Figure 1:**
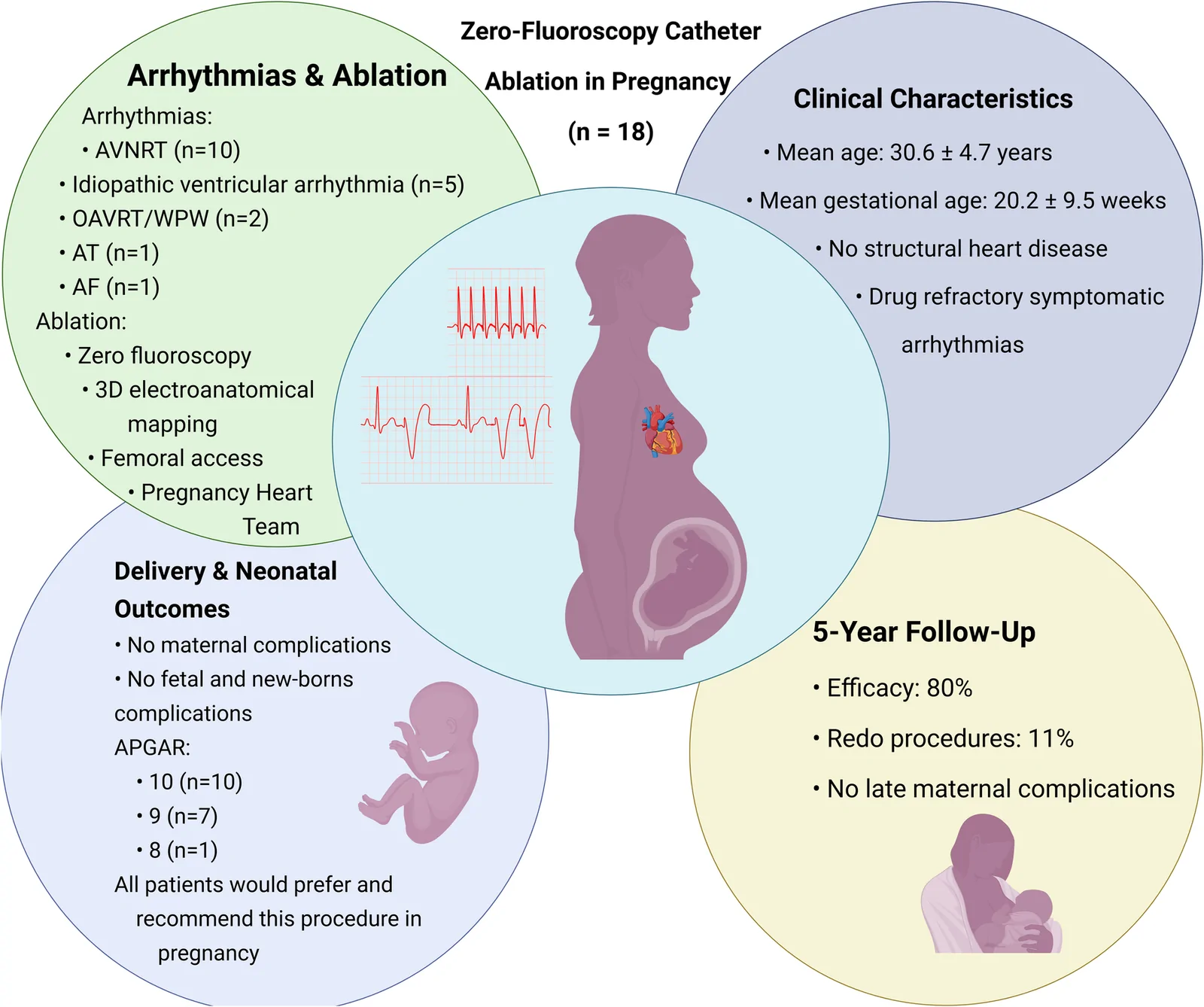
Major indications for catheter ablation in the study group.

The study group consisted of 18 pregnant women (mean age at the time of CA: 30.6 ± 4.7 years; range: 20–38 years; mean gestational age at the time of CA: 20.2 ± 9.5 weeks; range: 7–37 weeks). None of the women had overt structural heart disease. Based on data from the ELEKTRO-RARE-A-CAREgistry, pregnant women referred for a ZF-CA approach accounted for less than 0.01% of all cases; however, this incidence rose to 2% when analyzed exclusively among women of reproductive age. The main indications for CA were atrioventricular nodal reentrant tachycardia, focal idiopathic ventricular arrhythmia, orthodromic atrioventricular reentrant tachycardia in the course of Wolff–Parkinson–White (WPW) syndrome, atrial tachycardia, and AF (Figure 1). In three women, two arrhythmogenic substrates requiring CA were reported.

Detailed individual patient characteristics, procedural parameters, delivery data, and neonatal outcomes are summarized in Table 1.

**Table 1:** Clinical, procedural, and perinatal outcomes in pregnant women undergoing zero-fluoroscopy catheter ablation.

No	Maternal age (years)	Gestation (week)**/**gravidity	Type of arrhythmia	Procedure time min)	Delivery mode/gestation week	Apgar score	Child development	Second procedure needed	Antiarrhythmic drugs after CA
1	34	33/2	AVNRT	54	CS/36	10	Normal	No	No
2	20	20/2	AVRT^d^	60	CS/26	10	Normal	No	No
3	31	12/2	AVNRT	48	NC/40	10	Normal	No	No
4	30	36/2	AVRT^c^	48	CS/38	10	Normal	No	No
5	28	37/2	AVNRT	59	CS/38	10	Normal	No	No
6	32	31/2	AVNRT	54	CS^e^/39	10	Normal	Yes	No
7	33	17/6	AVNRT/PVC^a^	52	CS/40	9	Normal	No	No
8	32	7/1	PVC^a^	40	CS/38	9	Normal	No	No
9	32	11/4	AVNRT	41	CS/41	10	Normal	No	No
10	38	14/3	AVNRT	57	CS/37	9	Abnormal (delayed speech development, also present in their first child)	No	No
11	30	28/2	AVNRT	48	NC/36	9	Normal	No	No
12	35	24/2	PVC (2 foci)^b^	46	CS/37	10	Normal	Yes	No
13	29	7/1	PVC^a^	47	NC/38	10	Normal	No	No
14	27	23/2	AT	60	CS/37	9	Normal	No	No
15	34	23/2	PVC^b^	59	NC/41	9	Normal	No	No
16	29	10/2	AF	38	NC/37	10	Normal	No	No
17	37	20/3	AVNRT + AT	43	CS/40	10	Normal	No	No
18	20	11/2	AVNRT	55	NC/28	10	Normal	No	No

AT, atrial tachycardia; AVNRT, atrioventricular nodal reentrant tachycardia; CS, cesarean section; CA, catheter ablation; E, emergency childbirth; NC, natural childbirth; PVC, premature ventricular complex.

a RVOT (Right ventricular outflow tract).

b LVOT (Left ventricular outflow tract).

c Right-sided accessory pathway.

d Left-sided accessory pathway.

e Emergency childbirth.

A history of arrhythmia prior to pregnancy was present in 14 women (78%), and in three patients (17%) CA had been performed before pregnancy. During pregnancy, the most common manifestations of arrhythmia were palpitations (14/18; 78%), dyspnea (9/18; 50%), and syncope or near-syncope symptoms (7/18; 39%).

All procedures were successfully completed without fluoroscopy and without serious maternal or fetal complications. No vascular access complications were observed. The procedure time and ablation application time were 51.5 ± 6.8 min and 394 ± 338 s, respectively (Table 1). The median duration of the first follow-up assessment was 23.4 ± 13.6 months. Long-term telephone follow-up was performed 4–7 years after ablation (mean follow-up: 5 years). In one patient, a second procedure for idiopathic ventricular arrhythmia was postponed until after delivery. In another patient, arrhythmia recurred 2 months after delivery, requiring treatment with antiarrhythmic medication (beta-blocker) and a repeat CA procedure. However, in both cases, a significant clinical improvement after CA was observed and the arrhythmias were asymptomatic during pregnancy. The overall success rate of CA in the study group was 17/18 (94%) at 1-month follow-up, 15/18 (83%) at midterm follow-up, and 12/15 (80%) at long-term follow-up.

During follow-up after CA, one patient required emergency cesarean delivery for reasons unrelated to arrhythmia. In the remaining patients, no maternal complications were observed during the postpartum and postablation period. Among these patients, delivery occurred at 38 weeks of gestation in three cases, at 37 weeks in four cases, at 39 weeks in three cases, at 35 weeks in one case, and after 40 weeks in four cases. Five women had vaginal delivery and 10 underwent cesarean section.

No fatal complications were observed in the children. The Apgar score was 10 in 10 newborns, nine in seven newborns, and eight in one newborn. Child development was normal in all cases except for one child with speech problems, which were considered unlikely to be related to maternal arrhythmia during pregnancy or the CA procedure (Table 1).

During the long-term follow-up, no adverse effects related to ZF CA performed during pregnancy were observed in the children from the 15 families we were able to contact. One of the 15 women reported recurrence of arrhythmia during follow-up. Overall, after a mean follow-up of 5 years, CA performed during pregnancy remained effective in 80% of the women, enabling an asymptomatic and off-drug course until delivery.

## 4 Discussion

Our study represents one of the largest groups of pregnant women who underwent mapping and ablation using a standardized ZF-CA approach, without the use of intracardiac echocardiography and with very long-term follow-up. Only in one case of AF was transesophageal echocardiography required to guide ZF transseptal puncture and pulmonary vein isolation.

The majority of idiopathic maternal arrhythmias are benign and do not require invasive intervention. However, in selected patients with incessant and drug-refractory tachyarrhythmia, CA may be considered a rescue treatment. In these cases, a ZF approach represents an optimal treatment strategy during pregnancy. The Heart Rhythm Society expert consensus statement on the management of arrhythmias during pregnancy states that in patients with hemodynamically significant sustained arrhythmias refractory to or with contraindications to pharmacological therapy, CA may be considered. In such situations, the benefit of controlling maternal tachycardia should be prioritized over the potential radiation risks to the fetus, especially when the procedure is performed after the first trimester and radiation exposure is minimized according to the “as low as reasonably achievable” principle (4). Accordingly, the use of techniques and technologies aimed at minimizing radiation exposure during CA in pregnant women is recommended (4, 26). Catheter ablation can be potentially curative for SVTs of various mechanisms, including focal atrial tachycardia. Therefore, in pregnant patients with recurrent SVT refractory to pharmacological therapy or with contraindications to such therapy, CA is considered reasonable when strategies to eliminate or minimize radiation are applied (4).

In pregnant patients with tachycardia-induced cardiomyopathy, aggressive treatment of tachycardia is recommended, with beta-blockers as the first-line therapy and early consultation with an electrophysiologist for escalation of pharmacological therapy and/or consideration of CA (4, 6, 8–10). Current recommendations indicate that CA during pregnancy may be considered in women with hemodynamically unstable typical atrial flutter, recurrent hemodynamically unstable AF, atypical atrial flutter, or recurrent symptomatic or hemodynamically unstable ventricular tachycardia when pharmacological therapy is ineffective or contraindicated. In such cases, particular attention should be paid to the use of techniques that eliminate or minimize radiation exposure according to the “as low as reasonably achievable” principle (4, 6, 8–10).

In our registry, the most common indication for CA in pregnant women was atrioventricular nodal reentrant tachycardia, accounting for more than half of all ablation procedures. In routine clinical practice, this type of arrhythmia would be referred for CA without hesitation by the vast majority of cardiologists. However, during pregnancy, the decision becomes more complex because of the potential risk of fetal radiation exposure and periprocedural complications. Our data confirm previous reports that, in pregnant women, a ZF-CA approach performed after careful evaluation enables safe and effective treatment of maternal arrhythmias (1–3, 5, 7, 12–14, 19, 26, 27). In our registry, no CA-related maternal complications were observed, and the ZF-CA approach was not associated with adverse fetal outcomes. We also did not observe any negative impact of CA performed during pregnancy on Apgar scores or subsequent child development. Only one child was reported to exhibit delayed speech development. Notably, based on parental report, the same delayed speech development was observed in an older sibling, suggesting a familial predisposition rather than an adverse effect related to maternal arrhythmia or CA procedure. No congenital abnormalities, periprocedural complications, or adverse fetal events were identified. Therefore, no clinical evidence suggested a causal relationship between the catheter ablation procedure or periprocedural medications and the observed speech delay. Of note, none of the pregnant women undergoing CA in our registry had structural heart disease. In 78% of women, arrhythmia occurs prior to pregnancy; however, invasive treatment is performed before pregnancy in only 17% of cases. These findings highlight the need for increased vigilance regarding arrhythmia symptoms in young women of reproductive age and emphasize the importance of careful diagnostic evaluation. Once arrhythmia is confirmed, the potential role of CA should be considered to avoid the need for interventional treatment during pregnancy.

In contrast to other studies, a uniform CA protocol was applied in our registry, including consistent right femoral access, a two-catheter approach, and the use of the same recording system and catheter configuration. These factors, together with operator experience, resulted in relatively short procedure times. In the study by Koźluk et al. (12), different electroanatomical mapping systems and catheters were used, and atypical vascular access (a superior approach) for the ablation catheter was reported. Moreover, only 20.7% of the patients in the control group underwent the procedure using a ZF femoral approach. However, this study did not provide detailed procedural data that would allow comparison of the inducibility protocol or procedure duration.

In conference materials, Abdrakhmanov et al. (28) presented long-term results of ZF CA in a 2-year follow-up study. The authors analyzed outcomes in 48 pregnant women who underwent ZF CA. Similar to our study, the most common arrhythmia was atrioventricular nodal reentrant tachycardia (*n* = 21; 43.75%). Zero-fluoroscopy CA was performed under the guidance of CARTO (*n* = 21; 43.75%) and EnSite Precision systems (*n* = 27; 56.25%) (28). In our registry, the EnSite NavX system was used, which allowed visualization of catheters immediately after their insertion into the iliac veins, representing a potential advantage of this system. Our strategy also resulted in shorter procedure times (51.5 ± 6.8 min) compared with those reported by Abdrakhmanov et al. (71 ± 15 min) (28). The authors reported a procedure success rate of 100%. However, a procedural complication occurred in one patient (2.1%) who developed iliofemoral thrombosis, and two women experienced placental abruption and preeclampsia (4.2%) (28). No maternal adverse cardiac events were reported, similar to our population. The mean 5-min Apgar score was 8.4 ± 1.3 in their study (28), compared with 9.5 ± 1 points in our cohort. As in our study, no maternal or fetal mortality was observed.

In contrast to the study by Koźluk et al. (12), we did not encounter difficulties with catheter cannulation of the iliac veins or the superior vena cava. Owing to the standardized approach to coronary sinus cannulation using femoral access and the rapid diagnostic protocol—where coronary sinus cannulation was omitted after 5–10 min of unsuccessful attempts and arrhythmias were induced spontaneously using several maneuvers—the procedures were performed with high efficacy and relatively short procedure times.

Radiation exposure, because of its potentially harmful and sometimes unpredictable effects, is of particular concern in patients with special conditions such as pregnancy. The potential adverse effects of radiation may also affect the developing fetus and include organ malformations, intrauterine growth restriction, microcephaly, cognitive deficits, an increased risk of childhood cancer, and even fetal death (4, 6, 8–10, 29–31). Catheter ablation procedures are often avoided during pregnancy due to concerns about fetal radiation exposure. However, the risk of long-term malignancy in the fetus from the radiation doses typically required for these procedures is considered negligible (4).

Damilakis et al. (31) demonstrated that fetal radiation exposure during a standard EPS does not reach the threshold associated with a significant increase in lifetime cancer risk. In our study, the implementation of a standardized fluoroless protocol for CA in routine electrophysiological practice, combined with close collaboration with a pregnancy heart team, enabled safe and effective ablation of maternal SVT and idiopathic ventricular arrhythmias. The procedural success rate was 94% at short-term follow-up and 93% at long-term follow-up, with no adverse effects observed in the fetus or in subsequent child development after delivery. The high efficacy of the ZF-CA strategy, together with the very low risk associated with this invasive management approach, allowed avoidance of potential adverse effects of antiarrhythmic drugs and reduced the likelihood of arrhythmia recurrence during pregnancy, delivery, and breastfeeding in the majority of cases.

In this context, CA using three-dimensional navigation systems should be considered a last-resort therapy for life-threatening or drug-resistant arrhythmias in pregnant women. When CA is deemed necessary, the procedure should ideally be performed after the first trimester and, when possible, postponed to the third trimester, using minimal or zero fluoroscopy to reduce the risk of adverse fetal outcomes (4, 32). Techniques for reducing radiation exposure to both the mother and fetus include the use of three-dimensional mapping systems and intracardiac echocardiography (4–9). In our opinion, operator experience—particularly experience with a large number of ZF procedures—is also an important factor in minimizing procedural risks. In pregnant women undergoing high-risk CA procedures, care from a dedicated cardio-obstetrics team capable of managing potential complications, including urgent delivery if the fetus is close to term, is recommended (4, 33).

## 5 Limitations

This study has several limitations. Although the number of patients with structural heart disease was relatively small, the study cohort comprised all consecutive pregnant women referred to our electrophysiology center during the study period, reducing the risk of selection bias. In addition, all procedures were performed using a single electroanatomical mapping platform (Abbott), and therefore the findings may not be directly applicable to procedures performed with other mapping systems. Transseptal puncture was routinely performed under general anesthesia with transesophageal echocardiographic guidance. Where available, intracardiac echocardiography may represent an alternative imaging modality that could obviate the need for general anesthesia. Finally, all procedures were performed by operators with substantial experience in zero-fluoroscopy catheter ablation. Consequently, the safety and efficacy outcomes observed in this study may not be readily reproducible in centers with lower procedural volumes or less expertise in fluoroscopy-free techniques.

## Data Availability

The original contributions presented in the study are included in the article/Supplementary Material, further inquiries can be directed to the corresponding author.
